# Myopia in elementary school students in Eastern China during the COVID-19 pandemic

**DOI:** 10.3389/fpubh.2023.1167379

**Published:** 2023-06-21

**Authors:** Shuaishuai Huang, Fanhan Shen, Fujun Zhou, Qinghai Gong, Kui Liu, Wei Feng, Dong Cen

**Affiliations:** ^1^Ningbo Yinzhou No.2 Hospital, Ningbo, China; ^2^Fenghua District Center for Disease Control and Prevention, Ningbo, China; ^3^Ningbo Municipal Center for Disease Control and Prevention, Ningbo, China; ^4^Zhejiang Provincial Center for Disease Control and Prevention, Hangzhou, China

**Keywords:** COVID-19, primary school, influencing factor, myopia, pupil

## Abstract

**Background:**

Myopia is an increasingly serious public concern, particularly among primary school students. The prevalence of myopia and its influencing factors in primary school pupils in Eastern China during the COVID-19 pandemic had not been explored.

**Methods:**

A randomly clustered sampling method was performed, and selected pupils from grade 1 to grade 3 in 15 primary schools in the Fenghua District of Zhejiang Province were included and given myopia screening and uniform questionnaire survey 1 year later.

**Results:**

A total of 4,213 students completed the myopia screening and questionnaire survey. Myopia was diagnosed in 1,356 pupils, with a myopia incidence of 32.19%. The spherical equivalent (SE) refraction of the included pupils decreased on average by 0.50 ± 2.15 D 1 year later. The myopia rate was positively correlated with the increase of grade, in which the myopia rate among grade 3 students was the highest at 39.69%. The myopia rate among female students was higher than that among male students. Students residing in urban areas had a higher myopia rate than in rural areas. Maintaining an near work distance ≥33 cm was a significant protective factor (OR = 0.84, 95% CI: 0.74–0.96). Students with two myopic parents had a higher risk of myopia (OR = 1.61, 95% CI: 1.34–1.92).

**Conclusion:**

During the COVID-19 pandemic, the myopia rate among early primary school students in Eastern China was high. More attention and implementation of interventions from health and education departments, such as training the development of good eye behavior, should be considered to strengthen the intervention of myopia in primary school students.

## 1. Introduction

In recent years, the prevalence of myopia has increased markedly and rapidly, resulting in a serious burden among the general population (1–6). Its prevalence in East Asian countries, particularly in the Chinese population, is the highest (7). The prevalence of myopia among senior high school students has reached 80% in China, and that there is a trend of increasingly younger age at diagnosis (8, 9). Moreover, meta-analysis results have shown that the myopia rate among primary students in China was 22.53% during 1989–2014, whereas it has increased to 38.92% during 2018–2020, implying an increasing challenge for myopia's interventions (10, 11). Individuals who developed myopia during childhood were prone to developing high myopia, which could increase the risk of macular degeneration, retinal detachment, glaucoma, and cataract (12–15). These complications might further cause low vision and even blindness. Thus, how to curb children's myopia effectively has become an important issue of public health.

School-age children and adolescents have been seriously affected by the worldwide outbreak of COVID-19 from the end of 2019. Due to the indispensable quarantine measures required during the outbreak, children had less opportunities for outdoor activities, which inevitably implied increased time involved in online learning and other online activities (16). Previous studies have reported that the amount of time spent in outdoor activities and the amount of time spent reading at a close distance were two main factors that strongly influence myopia (17, 18). Although COVID-19 had no direct influence on myopia, it might have had an indirect effect via these factors, and therefore could have contributed to the prevalence of myopia. Hence, assessing these factors in Chinese children during COVID-19 pandemic was worth investigating.

The aim of this study was to explore myopia prevalence in primary school pupils in Eastern China during the COVID-19 pandemic. Fenghua district of Zhejiang Province was selected as the site to examine the potential influencing factors that would be helpful for the development of positive intervention for prevent myopia in this special group**.**

## 2. Materials and methods

In this study, Fenghua district was selected as the study site. Fenghua district is located in the coastal area of East China. It is between 29°25′ and 29°47′ north latitude, and 121°03′ and 121°46′ east longitude, and covers a land area of 1,277 km^2^. There are eight communities and four towns under the jurisdiction of Fenghua District, with 577,505 permanent residents. In Fenghua district, there are 30 primary schools, with nearly 30,271 pupils from grade 1 to 6.

### 2.1. Respondents

**Figure 1:**
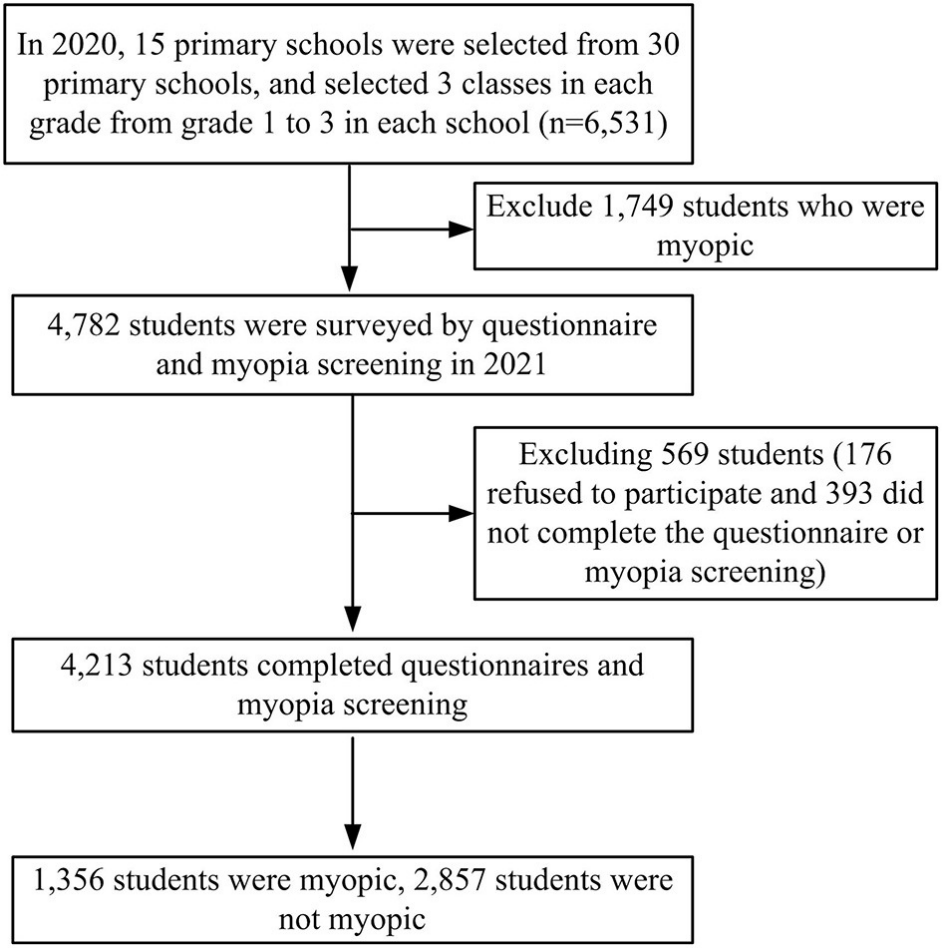
The flow chart of myopia screening among students.

A randomly clustered sampling method was used in this study in 2020. Firstly, 30 random numbers were assigned to 30 primary schools. Then, by randomly drawing 15 numbers, the specific numbers corresponding to schools were obtained. Subsequently, the same method was used to select 3 classes in each grade from grade 1 to 3 in each school. A total of 6,531 students were considered initially. From these students, 1,749 students who were already myopic prior to the study were excluded; thus, 4,782 students were included as research subjects. Questionnaires and myopia screening were carried out among these students 1 year later. Among the 4,782 included students, 4,213 students completed both the questionnaire survey and the myopia screening, with a completion rate of 88.10% (569 students were excluded: 176 refused to participate and 393 did not complete the questionnaire survey or myopia screening). The baseline data of SE refraction for the general characteristics of included participants in 2020 was presented in the Supplementary material 1. The electronic questionnaire included items on demographic characteristics, parent's myopia status, near work distance, average outdoor activity time, time spent in homework, average time spent on electronic device, and average sleep duration (Supplementary material 2). It was delivered using the student health management platform (SHMP) of Fenghua District. By scanning a QR code on this platform, students would complete the survey along with their parents by using mobile phones or other electronic equipment. The details of this process are shown in Figure 1.

### 2.2. Definitions

Myopia screening included an uncorrected distance visual acuity (UDVA) examination and refractive examination. The UDVA examination employed a standard logarithmic visual acuity chart, in which students were examined in the order of the right eye first, followed by the left eye, with an eye mask on the alternate eye, at a distance of 5 meters from the visual acuity chart. The results were recorded to one decimal place. Additionally, refractive examination was performed by means of a computer refractometer under non-cycloplegic conditions, and the average value of three measurements by a local ophthalmologist was taken. The combination of measurement indexes included spherical refraction and cylindrical refraction. Spherical equivalent refraction (SE) was calculated as spherical refraction + 1/2 cylindrical refraction. The criteria for myopia were set as UDVA < 5.0 and SE < −0.5 D. If a single eye was denoted as myopic, the participant was identified as having myopia.

### 2.3. Ethics statement

This study was approved by the ethics committee of Ningbo Yinzhou No.2 hospital. A standard informed consent section describing the research target was presented at the beginning of the electronic questionnaire. All personal information in this study was kept confidential as required and the research process complied with the Declaration of Helsinki.

### 2.4. Statistical analysis

Quantitative data are presented as mean (standard deviation). Considering the high correlation between the SE of the left eye and the right eye in students (*r* = 0.84), only the SE of the right eye was analyzed in this study. The SE among students with different variables were compared by *t*-test or analysis of variance. The chi-square test was used to evaluate factors influencing students' myopia. The variables with statistically significant differences were further considered in a multivariate logistic regression analysis. *P* < 0.05 was considered as indicating a statistically significant difference. All data were analyzed by using R v4.0.5 software (https://www.r-project.org/).

## 3. Results

### 3.1. The general epidemiological characteristics of myopia among students

In 2020, based on the principles of sampling and inclusion, a total of 4,782 pupils were recruited in this study. One year later, 4,213 pupils had ultimately completed the questionnaire and myopia screening, including 2,228 male students (52.88%) and 1,985 female students (47.12%). The myopia rate in students with different characteristics is shown in Table 1. The distribution of pupils overall, from grade 1 to grade 3, was as follows: 1,236 (29.34%) in grade 1, 1,609 (38.19%) in grade 2, and 1,368 (32.47%) in grade 3. There were 2,658 (63.09%) students residing in urban areas and 1,555 (36.91%) students residing in rural areas. Among 4,213 pupils, myopia was found in 1,356 students, constituting a myopia incidence of 32.19% with 95% CI 30.78%-33.60% (males: 30.66% and females: 33.90%, which was statistically significantly different; *P* < 0.01). Furthermore, the myopia rate showed a significant increasing trend with the increase in grade level (*P* < 0.01). The students living in urban areas had a higher myopia rate than did those in rural areas (*P* < 0.01). Students whose near work distance ≥33 cm had a comparatively lower myopia rate than those with a near work distance <33 cm (*P* < 0.01). Moreover, students whose parents had myopia appeared to have a higher risk of myopia progression (*P* < 0.01).

**Table 1:** The comparison of myopia incidence among students with different characteristics in Fenghua District.

**Characteristics**	**Number of students**	**Students with myopia**	**Myopia rate (%)**	**χ^2^**	***P-*value**
**Sex**					
Male	2,228	683	30.66	5.10	0.02
Female	1,985	673	33.90		
**Grade**					
Grade 1	1,236	289	23.38	79.32	< 0.01
Grade 2	1,609	524	32.57		
Grade 3	1,368	543	39.69		
**Place of residence**					
Urban area	2,658	918	34.54	18.24	< 0.01
Rural area	1,555	438	28.17		
**Parent's myopia status**					
Both parents without myopia	1,781	490	27.51	36.67	< 0.01
One parent with myopia	1,552	526	33.89		
Both parents with myopia	880	340	38.64		
**Near work distance**					
< 33 cm	1,776	617	34.74	9.18	< 0.01
≥33 cm	2,437	739	30.32		
**Average outdoor activity time**					
< 2 h	2,988	961	32.16	< 0.01	0.96
≥2 h	1,225	395	32.24		
**Average time spent in homework**					
< 1 h	2,069	684	33.06	1.42	0.23
≥1 h	2,144	672	31.34		
**Average time spent on electronic device**					
< 1 h	3,442	1,096	31.84	1.02	0.31
≥1 h	771	260	33.72		
**Average sleep duration**					
< 9 h	1,418	456	32.16	< 0.01	0.98
≥9 h	2,795	900	32.20		

### 3.2. Multivariate analysis of factors influencing myopia among primary school students

The factors that were identified as significantly influencing myopia in univariate analysis, including place of residence, parent's myopia status, sex, grade, and near work distance, were considered in the multivariate logistic regression analysis (Table 2). The myopia rate of the grade 3 students was found to be 2.18 times than that of grade 1 students. Students whose parents had myopia showed a higher risk of myopia occurrence [odds ratio (OR) = 1.61, 95% confidence interval (CI): 1.34–1.92], while near work distance ≥33 cm was a protective factor against myopia development (OR = 0.84, 95% CI: 0.74–0.96). Female had a higher risk of myopia progression than male. Students living in rural areas had a lower risk of myopia than those living in urban areas.

**Table 2:** Multiple logistic regression analysis of influence factors of students' myopia.

**Independent variables**	***X* ^2^**	***P-*value**	**OR**	**95%CI**
**Sex**				
Male			1	
Female	7.02	0.01	1.20	1.05–1.36
**Grade**				
Grade 1			1	
Grade 2	28.37	< 0.01	1.59	1.34–1.88
Grade 3	78.19	< 0.01	2.18	1.84–2.60
**Place of residence**				
Urban area			1	
Rural area	6.19	0.01	0.84	0.72–0.96
**Parent's myopia status**				
Both parents without myopia			1	
One parent with myopia	11.10	< 0.01	1.29	1.11–1.51
Both parents with myopia	27.12	< 0.01	1.61	1.34–1.92
**Near work distance**				
< 33 cm			1	
≥33 cm	6.70	0.01	0.84	0.74–0.96
**Average outdoor activity time**				
< 2 h			1	
≥2 h	< 0.01	0.98	1.00	0.87–1.16
**Average time spent in homework**				
< 1 h			1	
≥1 h	3.72	0.05	0.88	0.76–1.00
**Average time spent on electronic device**				
< 1 h			1	
≥1 h	1.30	0.26	1.11	0.93–1.32
**Average sleep duration**				
< 9 h			1	
≥9 h	0.01	0.91	0.99	0.86–1.14

### 3.3. The change value in spherical equivalent of pupils

During the study period, the pupils' SE of the right eye decreased with an average of 0.50 D, in which the variation of SE was 0.47 D in males and 0.52 D in females. By analyzing the differences in the three grades, we found a maximum variation (0.57 D) in students in grade 3. Differences in the SE of students with different characteristics are displayed in Table 3.

**Table 3:** The comparison of right eye's spherical equivalent of students with different characteristics between 2020 and 2021.

**Variables**	**SE, mean (SD) in 2020**	**SE, mean (SD) in 2021**	***T*-value**	***P-*value**
**Sex**				
Male	−0.67 (1.47)	−1.14 (1.56)	10.40	< 0.01
Female	−0.62 (1.39)	−1.14 (1.67)	10.74	< 0.01
**Grade**				
Grade 1	−0.69 (1.04)	−1.10 (1.47)	6.89	< 0.01
Grade 2	−0.61 (1.47)	−1.11 (1.61)	9.27	< 0.01
Grade 3	−0.64 (1.37)	−1.21 (1.68)	9.61	< 0.01
**Place of residence**				
Urban area	−0.68 (1.44)	−1.16 (1.65)	11.31	< 0.01
Rural area	−0.59 (1.42)	−1.12 (1.56)	9.85	< 0.01
**Parents' myopia**				
Both parents without myopia	−0.59 (1.46)	−1.12 (1.59)	10.29	< 0.01
One parent with myopia	−0.66 (1.40)	−1.13 (1.67)	8.59	< 0.01
Both parents with myopia	−0.72 (1.42)	−1.20 (1.57)	6.66	< 0.01
**Near work distance**				
< 33 cm	−0.66 (1.42)	−1.14 (1.62)	9.20	< 0.01
≥33 cm	−0.63 (1.40)	−1.14 (1.61)	11.81	< 0.01
**Average outdoor activity time**				
< 2 h	−0.67 (1.44)	−1.12 (1.64)	11.81	< 0.01
≥2 h	−0.57 (1.40)	−1.18 (1.55)	10.20	< 0.01
**Average time spent in homework**				
< 1 h	−0.65 (1.40)	−1.15 (1.62)	10.68	< 0.01
≥1 h	−0.64 (1.40)	−1.13 (1.61)	10.45	< 0.01
**Average time spent on electronic device**				
< 1 h	−0.63 (1.41)	−1.15 (1.63)	14.08	< 0.01
≥1 h	−0.69 (1.51)	−1.09 (1.52)	5.17	< 0.01
**Average sleep duration**				
< 9 h	−0.61 (1.45)	−1.11 (1.59)	8.68	< 0.01
≥9 h	−0.66 (1.42)	−1.16 (1.62)	12.16	< 0.01

## 4. Discussion

In recent years, the problem of myopia has increasingly received attention, given the increase in the myopia rate among younger students (2, 19–21). Our study showed that 32.19% of pupils were newly diagnosed with myopia, while the SE of pupils from grades 1 to 3 decreased by a mean of 0.50 D during the COVID-19 pandemic after a 1-year follow-up. One previous study performed in the Shanxi Province of China identified that the rate of myopia development of pupils in grades 1 to 3 was 20.05% within half a year during the COVID-19 pandemic (22). Another study in the Shanghai area reported that the SE among students aged 7–12 years decreased by an average of 0.59 D between April 2019 and May 2020 (23). Similarly, one investigation in Beijing showed that the SE of students aged between 8 and 10 years decreased by 0.60 D in 1 year during the COVID-19 pandemic (24). A meta-analysis of eight studies assessed the change in adolescent myopia development and compared the visual acuity before and after the COVID-19 pandemic. The review found a 0.41 D reduction in SE, which implied a negative effect of the pandemic on adolescent vision development (25). Simultaneously, studies in Turkey, Spain, and India reported similar conclusion (26–28). Although no strict before and after comparisons were performed in our study, the results were in line with other contemporary studies above, implying that an increased risk of myopia during the COVID-19 pandemic.

According to the findings of our investigation, the risks of myopia in students whose parents both had myopia and that of those who had one parent diagnosed with myopia were 1.61 and 1.29 times higher than those whose parents were both without myopia, respectively, indicating that myopia is influenced by genetic factor. A previous study analyzed the data of 15,316 students aged between 6 and 18 years in 19 schools in China, and concluded that the adjusted OR value of students whose parents both had myopia was 2.83, as compared to those whose parents both were without myopia (29). A multivariate analysis of factors affecting myopia, conducted in 16,771 students aged between 7 and 18 years in Beijing, concluded that the occurrence of myopia was positively correlated with parents' myopia status, with an OR value of 1.35 (30). Therefore, students with a single parent or both parents suffering from myopia should be treated as target groups of myopia prevention.

Our investigation demonstrated that a near work distance ≥33 cm is a potential protective factor for myopia. The cohort study conducted in Taipei followed 10,743 children aged 9 to 11 years over 2 years. They found that students with a near work distance >30 cm had significantly less myopic progression (31). Two other studies show that shorter working distance is related to higher incidence of myopia (32, 33). Therefore, one of the vital interventions is to develop good eye habits and conscious eye care behaviors.

As primary school age is a crucial period for myopia prevention, it would be appropriate that schools and parents should work together to guide students to develop good eye habits. Myopia among female students is higher than that among male students, which may be due to girls' shorter outdoor activity time, and consequent longer time for study (19, 34). Therefore, additional attention should be paid to female primary school students for myopia prevention and control. Additionally, the increased learning burden and increased time spent on studying could also explain the positive correlation between the myopia rate and increasing grade levels. Hence, it is necessary to persist in and reinforce interventions.

Our investigation also demonstrated that the myopia rate among pupils in urban areas is higher than that in rural areas, which was consistent with the data from other studies conducted in Shanxi province, Wuhan city and so on (35, 36). This difference might be attributable to potential differences in education pressure. Students in urban areas face higher academic pressure from school and family than students in rural areas, while students in rural areas have more opportunities to enjoy outdoor time. Moreover, electronic devices are increasingly popularized in urban areas, and better financial conditions in urban families are accompanied by a higher frequency of using electronic devices such as mobile phones and computers (37). Thus, decreasing the academic burden, encouraging more physical training and outdoor activity, and reducing the utilization of electronic devices in school children remain a priority.

Our study survey found no statistically significant correlation between daily outdoor time and myopia, which conflicted with other findings. One follow-up study in Britain showed that the longer the outdoor activity time of 3–9-year-old children, the lower the myopia prevalence was by the time these children grew up to be 10–15 years of age (38). Besides, another study illustrated that outdoor activity time was negatively correlated with the occurrence of myopia in both 6-year and 12-year age groups (39). In China, a cohort study in Guangdong Province presented that reduced outdoor activity was related to preschool myopia among 1–3-year-old children (40). The other cohort study in northeast China revealed that 20 min of additional outdoor activity every morning and every afternoon could obviously reduce the myopia levels in the intervention group (41). Given the potential home quarantine for all participants and the limited follow-up time, the effect of daily outdoor time might not be easily observed in our study. Nevertheless, we advocate for ensuring sufficient outdoor time to protect the eyesight of teenagers effectively.

Previous study demonstrated that students spend about 4.37 h/day on electronic device during periods of COVID-19 isolation, showing a positive relationship between myopia development and its used time (24). However, our finding did not identify an obvious association between electronic device use and myopia occurrence, which might be contributed to the cancellation of online education during our survey, that may cause the bias for this variable. Considering an increasing association between the used time of electronic device and myopia occurrence, it was still suggested that a wide health education should be performed among patients to reduce electronic device use in pupils.

SE was not only one of the indicators of myopia, but was also an evaluation index for hyperopia reserve. With appropriate hyperopia reserve, the occurrence of myopia among pupils, particularly those in low grades, could be slowed down. Although we did not perform a comparison in our study group, our results showed a mean difference of 0.5 D from grade 2 to grade 3 pupils, whereas this was only 0.31 D among the same age group in the Guangzhou study that was conducted before the COVID-19 pandemic (42). This implied that the risk of developing myopia was greater among students during the COVID-19 epidemic. Thus, health education and target interventions should be emphasized among primary school children to ensure that they have an adequate hyperopia reserve. This will undoubtedly benefit the prevention and control of myopia.

Some limitations of the study should be noted. First, since the diopter examination in the myopia screening was not performed under cycloplegic conditions, the observed myopia rate and the real rate may have differed. Second, we did not collect data on myopia before 2020, which might limit further analysis of the change trend before and after COVID-19.

## 5. Conclusion

In this study, the myopia rate among primary school students in Eastern China was high during the COVID-19 pandemic. The myopia rate among female students was higher than that among male students, and that among students living in urban areas was higher than that among students living in rural areas. Additionally, students with near work distance <33 cm, and those who had parents with myopia, were at greater risk of developing myopia. Thus, further attention and implementation of interventions, such as development of good eye habits, from health and education departments, are needed particularly in primary school students.

## Data availability statement

The original contributions presented in the study are included in the article/Supplementary material, further inquiries can be directed to the corresponding authors.

## Ethics statement

The studies involving human participants were reviewed and approved by the Ethics Committee of Ningbo Yinzhou No.2 hospital. Written informed consent from the participants' legal guardian/next of kin was not required to participate in this study in accordance with the national legislation and the institutional requirements.

## Author contributions

SH: writing—original draft, methodology, software, and funding acquisition. FS: data collection, investigation implementation, validation, formal analysis, and supervision. FZ: investigation implementation, software, and formal analysis. QG: supervision and methodology. KL: writing—review and editing and conceptualization. WF: visualization, software, conceptualization, funding acquisition, and supervision. DC: methodology and writing—review and editing. All authors contributed to the article and approved the submitted version.
