# Supplementary material for: Myopia in elementary school students in Eastern China during the COVID-19 pandemic
Source: Front Public Health. 2023 Jun 21;11:1167379. doi: 10.3389/fpubh.2023.1167379 (PMC10320216; doi:10.3389/fpubh.2023.1167379)
Supplement: Supplementary file 1 [file Table_1.doc]

**Supplement file 1**. General characteristics of included students’spherical equivalent (SE) refraction in 2020.

| Variables | SE refraction, Mean (SD) | T value | *P* value |
| --- | --- | --- | --- |
| **Sex** |  |  |  |
| Male | -0.67 (1.47) | -1.08 | 0.28 |
| Female | -0.62 (1.39) |
| **Grade** |  |  |  |
| Grade 1 | -0.69 (1.04) | 1.07 | 0.34 |
| Grade 2 | -0.61 (1.47) |
| Grade 3 | -0.64(1.37) |
| **Place of residence** |  |  |  |
| Urban area | -0.68 (1.44) | -1.91 | 0.06 |
| Rural area | -0.59 (1.42) |
| **Parents’ myopia** |  |  |  |
| Both parents without myopia | -0.59 (1.46) | 2.50 | 0.08 |
| One parent with myopia | -0.66 (1.40) |
| Both parents with myopia | -0.72 (1.42) |
| **Near work distance** |  |  |  |
| <33 cm | -0.66 (1.42) | -0.72 | 0.47 |
| >33 cm | -0.63 (1.40) |
| **Average outdoor activity time** |  |  |  |
| < 2 hours | -0.67 (1.44) | -2.02 | 0.04 |
| ≥ 2 hours | -0.57 (1.40) |
| **Time consumption of homework** |  |  |  |
| < 1 hour | -0.65 (1.40) | -0.23 | 0.82 |
| ≥ 1 hour | -0.64 (1.40) |
| **Average time spent on electronic device** |  |  |  |
| < 1 hour | -0.63 (1.41) | 0.98 | 0.33 |
| ≥ 1 hour | -0.69 (1.51) |
| **Average sleep duration** |  |  |  |
| < 9 hours | -0.61 (1.45) | 1.12 | 0.26 |
| ≥ 9 hours | -0.66 (1.42) |
