# Supplementary material for: Myopia in elementary school students in Eastern China during the COVID-19 pandemic
Source: Front Public Health. 2023 Jun 21;11:1167379. doi: 10.3389/fpubh.2023.1167379 (PMC10320216; doi:10.3389/fpubh.2023.1167379)
Supplement: Supplementary file 2 [file Table_2.docx]

Dear Parents/Guardians:

In order to understand the current situation of myopia-related behaviors of primary school students in Fenghua district, the Fenghua District Center for Disease Control and Prevention will conduct a survey on some students in grades one to three. The survey mainly includes demographic characteristics of students, myopia status of parents, eye-use behavior, electronic device usage, outdoor activities and sleep time. This survey uses an electronic questionnaire, and the students participating in the survey complete the questionnaire with their parents on their mobile phones by scanning the QR code to fill in. Your active participation will help us understand the current situation of myopia-related behaviors of primary school students in our district providing an important reference for government departments to formulate policies and measures to protect primary school students from myopia. The data of questionnaire will be analyzed anonymously, and the data will only be used for scientific research and cannot be shared with third parties. Of course, you can also refuse to participate in this survey. If you have any doubts, you can consult the Fenghua District Center for Disease Control and Prevention, we will patiently answer your question. If you agree, we will start the following questionnaire.

**Special investigation of students' eyesight situation and influencing factors**

**1 Essential information**

| **Item** | **Investigative content** | **Option** |
| --- | --- | --- |
| 1.1 | School |  |
| 1.2 | Grade |  |
| 1.3 | Surname and personal name |  |
| 1.4 | Sex | □ Male □ Female |

**2. Investigative Questions**

| **Item** | **Investigative content** | **Option** |
| --- | --- | --- |
| 2.1 | Was your school located in an urban or rural area? | □ Urban area □ Rural area |
| 2.2 | How long was the averaged homework after school in the past one week? | □ Less than 1 hour  □ 1-2 (excluding 2) hours  □ 2-3 (excluding 3) hours  □ 3 hours and more  □ Don't know  □ No homework |
| 2.3 | When you read and write, were your eyes more than a foot from the book (33 cm, about two small water bottles long)? | □ Never  □ Occasionally  □ Often  □ Always |
| 2.4 | Over the past one week, how much time did you spend on electronic device for one day? | □ I didn't use it before  □ Less than 1 hour  □ 1-2 (excluding 2) hours  □ 2-3 (excluding 3) hours  □ 3-4 (excluding 4) hours  □ 4 hours and more |
| 2.5 | How much outdoor activity time did you have during the day in the past one week (summary time)? | □ Less than 1 hour  □ 1-2 (excluding 2) hours  □ 2-3 (excluding 3) hours  □ 3 hours and more  □ Don't know |
| 2.6 | How long did you sleep every day on average? | Hours |
| 2.7 | were your parents nearsighted? | □ Only my father is myopic  □ Only the mother is myopic  □ Both parents are myopic  □Neither parent is nearsighted |
